# Ethics round table: choice and autonomy in obstetrics

**DOI:** 10.1136/jme-2024-110503

**Published:** 2024-11-29

**Authors:** Dominic Wilkinson, Safoora Teli, Claire Litchfield, Anna Madeley, Brenda Kelly, Lawrence Impey, Rebecca CH Brown, Elselijn Kingma, Helen Lynne Turnham

**Affiliations:** 1 John Radcliffe Hospital, Oxford University Hospitals NHS Foundation Trust, Oxford, UK; 2 Uehiro Oxford Institute, University of Oxford, Oxford, UK; 3 Murdoch Children's Research Institute, Melbourne, Victoria, Australia; 4 Centre for Biomedical Ethics, National University of Singapore Yong Loo Yin School of Medicine, Singapore; 5 Department of Paediatrics, School of Clinical Sciences, Faculty of Medicine Nursing and Health Sciences, Monash University, Melbourne, Victoria, Australia; 6 Oxford Maternal and Neonatal Voices Partnership, Oxford, UK; 7 Faculty of Health, Education & Society, University of Northampton, Northampton, UK; 8 Nuffield Department of Women's & Reproductive Health, University of Oxford, Oxford, UK; 9 Department of Philosophy, King's College London, London, UK

**Keywords:** Obstetrics, Personal Autonomy, Decision Making, Informed Consent, Ethics- Medical

## Abstract

Decisions about how and where they deliver their baby are extremely important to pregnant women. There are very strong ethical norms that women’s autonomy should be respected, and that plans around birth should be personalised. However, there appear to be profound challenges in practice to respecting women’s choices in pregnancy and labour. Choices carry risks and consequences—to the woman and her child; also potentially to her caregivers and to other women.

What does it mean for women’s autonomy to be respected in obstetrics? How should health professionals respond to refusals of treatment or requests for care outside normal guidelines? What are the ethical limits to autonomy? In this clinical ethics round table, service users, midwives, obstetricians, philosophers and ethicists respond to two hypothetical cases drawn from real-life scenarios.

## Cases^i^

### Case 1

Felicity is 44 years old and expecting her first baby. She has a history of anxiety and depression and a raised body- mass index of 40. Her pregnancy has been uncomplicated, though the baby is estimated to be large (>95th centile).

She is 39 weeks gestation today. She has been offered induction of labour at 40 weeks for maternal age.

Felicity wants minimal intervention during birth. She declines continuous fetal heart rate monitoring and would like to use a birthing pool for labour and delivery. She does not want an epidural for pain relief.

In discussion at today’s appointment, Felicity does not wish to take up the offer of induction of labour. She has heard that induction increases chance of interventions including emergency caesareans, is worried about hyperstimulation and/or it simply not working and ‘ending up’ with a caesarean anyway.

How should Felicity’s maternity team respond to her refusal of recommended care?

### Case 2

Rosa is 37 years old. She is 39 weeks pregnant in her second pregnancy. Her first birth experience was very traumatic. She previously laboured quickly, arriving at the midwifery -led unit when she was 8 cm dilated but transferred to delivery suite for slow progress in second stage. She went on to have an emergency caesarean section birth after failed attempt to delivery using forceps and major haemorrhage of 1800 mlL. She found her recovery from the birth prolonged, very painful, and her son was left with a small mark above his eye.

This pregnancy has been uncomplicated apart from anaemia which was treated with an iron injection and her haemoglobin is now just within the normal range at 105 g/lL.

Rosa is worried about impending birth. She found the previous birth frightening; she did not know what was happening and she thought she was going to die. She would like to have her baby at home this time and has requested a home birth. If this cannot be supported by the midwifery team, she would freebirth rather than be forced to go to hospital. She says she would not give permission for use of forceps or caesarean birth under any circumstances.

How should Rosa’s maternity team respond to her request for a home birth that might be associated with significant risk?

## Safoora Teli

### Lived experience/service user perspective

 For most individuals, choosing to birth ‘outside of guidelines’ is not a flippant decision. There may be significant experiences and extensive research behind their position. As an unconventional and sometimes difficult route that can involve disagreement from family, it can also feel confrontational, with expectations of having to ‘convince’ the clinical team and even consider disengaging from them.

In these scenarios, the team has an opportunity to dispel the notion that they are adversaries and to state their position as trusted professionals providing a safety net during pregnancy, birth and beyond.

The ultimate power for decision-making regarding birth lies with the individual. In summoning the courage to go ‘outside guidelines’, Rosa and Felicity have already recognised this. The teams should clearly acknowledge this agency too, thus demonstrating their meaningful and individual-centred approach.

All interaction should start with empathy. Accordingly, the teams should always be cognisant of the sensitivities regarding mental well-being of both women. Interaction that feels confrontational can heighten stress levels which is unhelpful in advanced pregnancy and may also trigger the fight or flight response, pushing them towards disengagement.

It is critical that the team does not use scare tactics, as this is an unsound basis for decision-making. Rosa had a traumatic experience in the hospital and was left feeling unsafe and vulnerable. Any information regarding the potential risks of her situation needs to be delivered sensitively. The conversation should begin with validation of her past experience. Following this, any mention of risks needs to come after the team has reiterated its key objective in supporting her for a safe and stress-free birth.

Ideally, Rosa should have been offered psychological support after her first birth. It may be inadvisable to revisit her trauma at this stage, but the team should offer appropriate resources and tools to support her mental health. They could use open questions to gauge her parameters, for example, would she consider attending the birthing unit at an earlier or later stage of her labour this time. If she is resolute in wanting a home birth, other considerations could be explored such as continuing to increase her iron levels and putting a cannula in place. She accepted an iron injection, which indicates an acceptance of interventions for an existent need.

While the team is focusing on mitigating risk, given the unknowns, there is a margin of variability; growth scans are not always accurate, and older mothers or those with a higher body mass index do not always have trickier labours. Similarly, induction can indeed lead to a cascade of interventions. As a first-time mother, there is no prior indication of how Felicity’s birthing experience might be. There is a real possibility that it may go smoothly. Either way, if she feels unheard and stressed, she will remember it and—like Rosa—carry it with her, potentially triggering her anxiety and depression, affecting her postpartum health, her motherhood journey and subsequent pregnancies.

Every individual is unique and encouraged to make a birth plan accordingly. She should feel her voice is central to the decision-making process, and not assumptions based solely on categories she falls into on paper. If the team provides positive support and advocacy without pigeonholing or dismissing her, it will build a foundation of trust. If any unplanned intervention is required, suggestions coming from the team will be seen as an extension of this support and be more welcome.

The teams should focus on what Rosa and Felicity are prepared to accept and not fixate on their refusals. Felicity is happy to be in a clinical setting and is more likely to accept necessary interventions if happy with her team. Rosa does not want to be in hospital but is willing to have midwives at her home birth. If she feels empathy from her team then she is more likely to trust them and consider a transfer to hospital if recommended.

Pressurising women in decision-making about birth is disempowering and leads to long-lasting trauma and distrust in maternity care. Conversely if at each stage of the process, they feel heard and supported to make their own choices, the experience will be both satisfying and empowering.

## Claire Litchfield, Anna Madeley

### Midwifery perspective

Contemporary guidelines and teaching encourage midwives to respect and support non-normative birth choices. However, whether midwives feel this in practice is debatable. Midwives described feeling fear of punishment and/or blame in the event of an avoidable poor outcome. There is potential for moral injury when midwives are presented with distressing physical situations at the outer limits of their professional scope of practice.

In case 1, midwives supporting Felicity may feel conflicted between supporting choices and their core role of optimising normal physiological processes.^1^ Currently, there is no guidance for midwives who wish to object to attending to women making non-normative birth choices and this could be an area for future discussion and debate.

Some National Health Service organisations provide birth options counselling, usually with a consultant midwife, possibly in a dedicated birth options clinic, where detailed plans can be made. These plans provide attendant midwives with explicit organisational support. Many plans seek to mitigate risks or maximise safety, which could involve negotiation with women, for example, midwives may offer Rosa (case 2) IV access during labour and the provision of drugs immediately after birth to reduce the chance of bleeding. Birth options clinics are recommended by National Institute for Health and Care Excellence, to provide counselling for women requesting caesarean birth without clinical indication^2^; however, in practice, they are also used to plan care for those making other non-normative choices.

If organisations do not provide birth options clinics or planning, midwives may feel more exposed personally and experience heightened feelings of fear. Where midwives are working in organisations which do not provide resources to support home births or midwifery units, or who prioritise strict criteria/compliance to clinical guidance, it may be practically impossible for midwives to attend women choosing non-normative care under the conditions of their employment. This may effectively force women to explore alternatives such as private maternity care, freebirthing or reluctant compliance.

From a legal and regulatory perspective, clinicians are required to provide person-centred care including where recommended care is declined or outside of clinical guidelines. Supporting women in their right to decline aspects of their care or make challenging choices is explicitly reflected in professional codes of conduct for clinicians.^1 3^ These protect clinicians from regulatory and legal action where core tenets of informed choice consent and supported decision-making are observed. Women are also enabled through legal precedent and statutory instrument to conduct their pregnancy and birth how and where they so please. Such protections include choosing with whom, the extent to which perinatal care is accepted (in whole, in part or not at all), and finally, and most critically, the ability not be compelled to yield bodily autonomy towards caregiver’s preference for actions or choice, merely by virtue of them disagreeing with a decision or by their decision be seen as risky, irrational or putting themselves or fetus in harm’s way.^ii^ Cases brought before the European and domestic courts reinforce these rights and emphasise the requirement to protect, through dynamic practices for obtaining informed consent and the assumption of mental capacity unless proven otherwise, the need to protect autonomy through the agency to exert choice and control. These matter to women^4^,^6^ informing immediate and ongoing decision-making whether that is declining recommended care, withdrawing from care altogether, or making increasingly non-normative choices. The dichotomy between legal rights towards choice, agency and autonomy and institutional actions and restrictions in care provision intended to influence compliance is well documented,^7^ as are the consequences of such choices for future and ongoing decision-making.^8^ These issues reinforce the nature of the challenge for midwives in facilitating complex person-centred choices, especially in the context of non-normative ones, as represented by this pair of cases.

## Brenda Kelly, Lawrence Impey

### Obstetric perspective

A person has both a moral and legal right to decline medical intervention and care, and caregivers have a duty to care for them as best they can, providing their care is accepted. Effective caregiving involves listening to the patient’s concerns, engaging with them and building trust.

Understanding the reasons behind a person’s labour and birth plan, including their hopes and fears, is crucial. It is important to assess their comprehension of the risks associated with deviating from recommended pregnancy and birth care, what level of risk they consider acceptable and which outcomes they value most. The decision-making process often involves trade-offs between what the mother deems optimal for her, whether related to physical or psychological well-being and the associated risks for the baby. Presenting the best available evidence in an understandable and applicable manner is central to informed decision-making.

Risk prediction in normal life events, such as childbirth, is imprecise, and many interventions might be needed to prevent a single adverse outcome like stillbirth. A respectful and psychologically safe space for discussions can help prevent disengagement from maternity care, which might inadvertently increase stillbirth rates. Interventions such as labour induction often prioritise fetal health over maternal health and can cause both physical and psychological trauma to the mother, impacting long-term well-being.

Felicity is at an increased risk of stillbirth beyond 40, and particularly 41–42 weeks of gestation, mainly due to her age. Although her pregnancy has thus far been uncomplicated, this risk is reduced but not eliminated. The absolute chance of stillbirth is low, around 1 in 100. While labour induction does not increase the risk of caesarean birth, it medicalises the labour experience and may make it less positive for the woman. Not inducing labour slightly increases the absolute risk of stillbirth, and it should also be acknowledged with Felicity that advancing gestation in a 44-year-old first-time mother may increase the likelihood of labour complications, such as fetal distress, even if labour begins naturally, potentially compromising her birth experience anyway.

Rosa’s traumatic previous birth influences her current pregnancy decisions. Working with Rosa to optimise her birth experience can help rebuild her psychological well-being. She has at least a 70% chance of achieving an uncomplicated vaginal birth, given her previous labour progress. A home birth, if appropriately supported, might offer her the best chance of a positive experience, as she would likely feel most relaxed. However, labour and birth can be unpredictable. Rosa has a 1 in 200 chance of her uterine scar from a previous caesarean section rupturing during labour. If this occurs at home, there is more than a one-in-two chance of stillbirth, with significant risk to her life from internal bleeding. Urgent transfer to the hospital would be necessary in order to maximise safety for her and her baby.

While maintaining hope is important for Rosa’s psychological well-being, it is crucial to discuss and agree on a contingency plan for potential complications, including when and why a transfer to the hospital would be recommended. Establishing whether she would consent to transfer on the advice of her caregivers is vital. These discussions might be profoundly triggering for Rosa, necessitating additional mental health support and follow-up conversations. If Rosa decides to have a home birth, she must be supported, as disengagement from care and freebirthing carry much greater risks. Caring for her at home will require resources and might further traumatise her and her caregivers in an emergency, as their assistance would be limited. Any advance directives against intervention should be revisited in case of an emergency at home.

Discussing non-normative birth choices requires training, empathy, experience and time, often involving multiple consultations. The needs of other patients and staff must also be considered, as dedicating time and resources to one patient could compromise the care of others, potentially creating conflict within a resource-constrained setting. Not all staff feel adequately experienced or psychologically safe to provide birth care outside standard guidelines. Traumatic experiences for staff might impact their ability to care for other mothers in the future.

In summary, balancing the rights and preferences of mothers with the duty of care requires sensitive, respectful communication and an understanding of the associated risks. Creating a supportive environment for informed decision-making and contingency planning is essential, even when dealing with non-normative birth choices. This approach ensures the well-being of both the mother and the caregivers, minimising trauma and maximising positive birth experiences.

## Rebecca CH Brown, Elselijn Kingma

### Philosophical perspective

The above cases can, understandably, be experienced as troubling by healthcare providers. That does not mean core ethical principles cease to apply. On the contrary: they are central to considering how best to support someone who wishes to deliver ‘outside the guidelines’.^iii^

Pregnant people, like any other competent adult, retain their near-absolute right to refuse medical treatment.^9^ In maternity contexts, autonomy is at once of special importance, and at particular risk of being undermined. Of special importance because maternity care (1) involves socially sensitive body parts and (2) often aims at promoting the health of one (the baby) at a cost to another (the mother).^10^ There is a particular risk of undermining the autonomy of labouring individuals, as it is often not adequately respected.^10^,^12^ The role of maternity care professionals in facilitating decision-making is not to coerce, cajole or manipulate people into making the ‘correct’ choice, but to enable the pregnant person to make genuinely autonomous decisions.

A key element of this is building rather than undermining trust. This requires supportive communication; a willingness to take the pregnant person’s concerns seriously; and consistent reassurance that their right to decide what is done to them will always be respected.

But in ‘outside guidelines’ cases, like the ones discussed here, healthcare providers are often concerned about the risk to the mother and the baby. How do healthcare providers best meet their ethical and professional obligations to provide safe and just care in such cases, all the while respecting women’s autonomy and building trust?

Draft Dutch guidelines, based on extensive ethical analysis, recommend that care providers take the steps outlined in box 1:

We can apply this to both Felicity and Rosa. In both cases, the care provider should take time to identify the underlying concerns (step 1). Why does Felicity want minimal interventions? What are her underlying beliefs, values and concerns? Only with an adequate understanding of these can the care provider give accurate, relevant and unbiased information about the pros and cons of induction (step 2). They can express concern about Felicity’s decision if they feel that is warranted, but only while making clear that they will respect what Felicity choses, and do their utmost to care for Felicity and her baby in any scenario (step 3). If Felicity continues to prefer not to induce, the care provider and Felicity should make a care plan for the safest possible care consistent with Felicity’s values and concerns. This should involve, for instance, discussing at what stage Felicity would consider induction, when this decision can be rediscussed, her preferences around intermittent auscultation (given a preference against continuous fetal heart rate monitoring) and so on (step 5). The plan needs to be clearly written down and communicated to the rest of the team that is (or is likely to become) involved in her care.

Box 1 **Recommendations for health professionals in responding to requests for care outside guidelines^15^**

1. Approach the request (or refusal) with an open frame of mind, taking care to identify the underlying concerns. Often concerns can be removed or accommodated in the context of good communication.
2. Give relevant unbiased information. This can include seeking to correct false beliefs and (sensitively) informing the pregnant person that they do not recommend what she is proposing.
3. Work with the pregnant person to identify the safest version of a care plan consistent with their wishes. Implement this plan, working to bring the entire care team on board.
4. Make clear the pregnant person can always change their mind, and check at regular intervals whether the plan needs changing (but not so often as to constitute bullying or nagging, or to undermine trust).
5. Carefully document that the plan deviates from medical recommendations, and why, as well as what has been agreed. This is to protect the health care provider and facilitate team collaboration.

For Rosa, too, the starting point must be to understand her background beliefs and values (step 1). Given Rosa’s previous negative experience, building trust and reassuring her that she will not be subject to interventions without her consent will be particularly important.^13 14^ Rosa’s care provider will need to ensure that Rosa is (sensitively, non-coercively) informed of the risks associated with her mode of delivery, such as uterine rupture, and that she prefers those risks over those associated with alternative birth settings (step 2). In developing a care plan (step 3), sensitive execution of steps 1 and 2 might open up avenues previously unavailable, such as an alongside midwifery unit or intermittent auscultation. This plan can be revisited and revised as appropriate (step 4) and should be shared with the entire care team (step 5). Where team members have reservations, they should be reassured that Rosa’s preferences and values have been carefully considered and the risks she faces explained to her. And also that by supporting an attended home birth (as opposed to a free birth), they are providing the best and safest achievable care for Rosa and her baby.^15^

Cases such as those of Felicity and Rosa may be troubling for healthcare providers. ‘Success’ in planning care for these women should not be measured against the extent to which they can be persuaded to comply with recommended care. Instead, it should focus on facilitating autonomous informed decisions, and on using the experience of the care teams to provide the best possible care for mother and baby consistent with those decisions.

## Dominic JC Wilkinson, Helen Turnham

### Clinical ethics

The above commentaries have already explored many of the important ethical considerations in the cases. In addition to those, if brought to our clinical ethics committee, we would aim to help clinicians in identifying and separating several distinct ethical questions.

### Autonomy and resources

The two cases in this paper represent two distinct ways in which autonomy challenges can arise. The first (as in case 1) is when patients refuse treatment offered. The second (case 2) is when patients request options that health professionals do not endorse or are not offering.

A standard ethical response to such challenges distinguishes between negative autonomy and the absolute right of patients to refuse treatment, versus positive autonomy and patients’ lack of a right to demand treatment (particularly where resources are scarce or that will negatively impact the care of other patients). But as the foregoing discussion makes clear, in practice the lines between positive and negative autonomy can be blurred. Refusal of treatment options can also impact resources and other patients, because such patients may need additional monitoring or alternative forms of care. And (as in the second case) requests for treatment may coincide with declining other treatments. Because Rosa is not willing to give birth in hospital, it is a mistake for clinicians to compare the options of hospital birth or home birth. The realistic options for her are either supported home birth or the much more risky freebirth.

We mentioned one potential limit to patient autonomy—that of scarce medical resources including physical (operating theatres, delivery suite space), personnel (staff time) and financial. But although resource limitations are an ethically important consideration they are difficult to apply to individual cases. That is for several reasons. First, unlike decisions about provisions of expensive drugs or organs for transplantation, allocation is not necessarily either/or, but how much of a resource should be offered. And it can be very difficult to draw a non-arbitrary line. Second, providing the desired resource may be feasible for an individual patient and will not necessarily lead to compromise in the care of other patients. The problem may arise when such cases occur repeatedly, as this can compromise the delivery of care to others. But it can be problematic to deny women access to treatment options that would be available to other women on such a basis (eg, the option of home birth or caesarean section). It is not simply a question of whether a resource is available, whether there is evidence supporting a choice, or even whether it is ‘cost-effective’. The real question is whether the benefit (eg, in terms of respecting a woman’s choices around birth) is sufficient to justify the provision of the requested resource. But that is a much more complicated question.

### Woman versus fetus

Next, one general constraint on patient autonomy is the potential for a choice to harm someone else. Choices about childbirth that fall outside guidelines might be thought to be particularly challenging because of the potential for harm to the fetus or future child. Of note, the above commentaries do not dwell on that particular question. That is because, at least in the UK context, the woman’s rights to make decisions about her own body and about childbirth, trump considerations of the well-being of the child. That is not to say that concern for the future child is ethically irrelevant.^16^ In most cases, such concerns will be at the front of the woman’s mind. They will likely also underpin the recommendations of midwives and obstetricians. However, we should be clear that this factor should not limit or constrain Felicity or Rosa’s choices. It would not be justified to force Felicity to have an induction of labour, or Rosa to give birth in hospital.

However, it can nevertheless be very important to openly discuss concerns about harm to the fetus/future child. That is because individual clinicians may have trained or worked in other parts of the world that limit women’s autonomy for the sake of the child. It is important to help them to understand how the approach may be different in countries like the UK. We might also explore whether a woman’s choices would conflict with the personal values and beliefs of the clinicians. Giving clinicians an opportunity to reflect on their own values can help to alleviate moral distress. It can also point to options that are available to them, including supporting the woman’s choice notwithstanding their personal disagreement,^17^ or the option of conscientious objection (where there are other clinicians available to support the woman in her care).

## Footnotes

These are hypothetical cases, though based on the clinical experience of the authors.

Re MB (Medical Treatment) [1997] EWCA Civ 3093, 1997; St George’s Healthcare NHS Trust v SR v Collins and others ex parte S, 1998

Unpublished Kingma, E. (2021) Toelichting Ethische Aspecten Verloskundige Zorvragen Buiten de Richtlijn. [‘Explanation of Ethical Aspects of Obstetric Care Requests Outside Guidelines’] (text available on request from author: elselijn.kingma@kcl.ac.uk)
